# Administration of Apple Polyphenol Supplements for Skin Conditions in Healthy Women: A Randomized, Double-Blind, Placebo-Controlled Clinical Trial

**DOI:** 10.3390/nu12041071

**Published:** 2020-04-13

**Authors:** Toshihiko Shoji, Saeko Masumoto, Nina Moriichi, Yasuyuki Ohtake, Tomomasa Kanda

**Affiliations:** 1 Research Laboratories for Fundamental Technology of Food, Asahi Breweries Ltd., 1-21 Midori 1-chome, Moriya-shi, Ibaraki 305-0106, Japan; saemail@agri.fukushima-u.ac.jp (S.M.); saito.nina@hata-da.co.jp (N.M.); yasuyuki.otake@asahi-gf.co.jp (Y.O.); tomomasa.kanda@asahigroup-holdings.com (T.K.); 2 Food Research Institute, National Agriculture and Food Research Organization, 2-1-12 Kannondai, Tsukuba-shi, Ibaraki 305–8605, Japan; 3 Faculty of Food and Agricultural Sciences, Fukushima University, 1 Kanayagawa, Fukushima-shi, Fukushima 960-1269, Japan; 4 Asahi Group Foods, Ltd., 2-4-1 Ebisuminami, Shibuya-ku, Tokyo 150-0022, Japan; 5 Asahi Group Holdings Ltd., 1-21 Midori 1-chome, Moriya-shi, Ibaraki 305-0106, Japan

**Keywords:** Apple polyphenols, procyanidin, UV irradiation, skin condition, pigmentation

## Abstract

This clinical study was performed to evaluate the effects of continuous apple polyphenol (AP) administration on facial skin conditions and pigmentation induced by ultraviolet (UV) irradiation in healthy women participants. Participants (n = 65, age 20–39 years) were randomized to receive tablets containing AP (300 or 600 mg/day) or placebo in a double-blinded, placebo-controlled clinical trial. Continuous administration of AP for 12 weeks significantly prevented UV irradiation induced skin pigmentation (erythema value, melanin value, L value), although a dose-dependent relationship was not clearly observed. In contrast, no significant differences were detected between the groups with regard to water content and trans-epidermal water loss. Our study demonstrated that APs and their major active compounds, procyanidins, have several health benefits. Here, we report that continuous administration of AP for 12 weeks alleviated UV irradiation induced skin pigmentation, when compared with placebo, in healthy women.

## 1. Introduction

The skin is an organ that safeguards and protects the body from harmful environmental factors such as pathogenic microorganisms, ultraviolet (UV) radiation, and temperature changes and prevents excessive moisture loss [1]. The skin is composed of three different structures: epidermis, dermis, and subcutaneous tissue, which facilitate the barrier functions described above [2,3]. However, the skin undergoes oxidative damage due to various stressors including daily UV exposure from the sun. Particularly, solar UV radiation elicits various adverse effects on skin tissue, including erythema, sunburned cells, inflammation, hyperplasia, hyperpigmentation, immunosuppression, photoaging, and even skin cancer.

Impaired skin structures result in increased trans-epidermal water loss (TEWL) and decreased water/moisture content in the skin. UV irradiation at low doses accelerates the melanogenesis of melanocytes in skin, whereas UV irradiation at high doses and repeated UV exposure induce carcinogenesis and photoaging of the skin. UV generates reactive oxygen species (ROS) and free radicals such as superoxide anion radical (O_2_^–^) and hydrogen peroxide (H_2_O_2_) [4,5]. UV irradiation and ROS increase matrix metalloprotease (MMP) expression and activity, which are responsible for degrading extracellular matrix proteins such as collagen and fibronectin. In addition, ROSs induce protein and lipid oxidation, cell membrane damage, and DNA damage including 8-hydroxy-2′-deoxyguanosine (8-OHdG) and thymine dimers [6]. Pyrimidine dimers and 8-OHdG are representative DNA base modified products generated by ROS in the skin [7,8]. In hyperpigmentation of melanocytes, H_2_O_2_ and thymine dimers are reported to increase the mRNA level of tyrosinase, a key enzyme during melanin biosynthesis in melanocytes [9,10]. ROSs are scavenged by protection systems, such as superoxide dismutase (SOD), catalase, and glutathione peroxidase. However, these enzymes are unable to completely scavenge and remove the ROSs and free radicals [11]. Therefore, prevention and protection from UV-mediated cellular damage may be useful for preventing skin disorders. To neutralize these ROSs, phytochemicals including polyphenols, carotenoids, and antioxidants in foods are required [6,12].

Dietary supplementation with vitamins, carotenoids, and phytochemicals such as polyphenols are proposed to improve skin conditions. Dietary supplementation with astaxanthin increased the minimal erythema dose (MED) and attenuated the UV-induced moisture loss in healthy humans [13,14,15]. Similarly, polyphenols are botanical secondary metabolites used as the main natural compounds in cosmetic applications [16]. Procyanidins, commonly known as polyphenols, are abundantly found in foods including fruits, vegetables, nuts, seeds, and bark, and beverages including red wine and cocoa [17]. Procyanidins have been suggested to have strong antioxidative activity [18,19] and various physiological functions [20,21,22,23,24], as demonstrated with in vitro molecular investigations and in vivo animal studies. Apples are widely consumed throughout the world and are an important source of procyanidins in the human diet [17,25]. Apples contain different structural subclasses, including phenolic acids and flavonoids, comprising six major classes: phenolcarboxylic acids (e.g., chlorogenic acid), anthocyanins (e.g., cyanidin glycosides), flavonols (e.g., quercetin glycosides), dihydrochalcones (e.g., phloridzin), flavan-3-ols (e.g., catechin), and procyanidins (e.g., procyanidin B2) (Figure S1). In our previous studies, we investigated the effects of APs on tyrosinase and anti-melanogenesis in B16 mouse melanoma cells, as well as the relationship between the procyanidin structure and its polymerization degree and anti-melanogenesis [26]. However, there are very limited human clinical trial data on the effects of apples and their polyphenols on skin pigmentation.

In the present study, we investigated whether orally administered AP could inhibit UV irradiation induced skin pigmentation in a human clinical trial. We carried out a randomized, double blinded, placebo-controlled trial to assess whether continuous administration of AP would affect the skin color and pigmentation in healthy women participants exposed to UV irradiation. Here, we demonstrate that AP administration for 12 weeks alleviated the pigmentation in the study participants, although a dose-dependent relationship was not clearly observed.

## 2. Materials and Methods

### 2.1. Study Participants

Healthy women participants (age 20–39 years) with skin photo-type-II and -III, registered with the Dermis Research Center Co. (Osaka, Japan), were recruited to the study. The trial was carried out for 12 weeks from the end of January to April. Participants with the following criteria were excluded from the study: (1) regular ingestion of antioxidative products and supplements including vitamin c, CoQ10, and polyphenol extracts such as grape seeds and green tea; (2) skin conditions including atopic dermatitis; (3) significant disease history of heart failure, dyslipidemia, diabetes, or uncontrolled hypertension; (4) risk of food allergy; (5) pregnant, lactating, or breastfeeding women; (6) extreme skin condition changes due to menstruation or irregular menstruation; (7) participation in other clinical trials at the start of this study. After receiving a full explanation of the study, all participants provided written informed consent. The participants were randomly assigned to three groups to receive (1) active 300 mg of AP supplement/day, (2) active 600 mg of AP supplement/day, or (3) placebo 600 mg starch decomposition product/day. The tablets were prepared and labeled by study staff in accordance with the randomized participant list.

### 2.2. Dietary Supplements

A commercial AP (Applephenon^®^, Asahi Breweries Co. Ltd., Tokyo, Japan) was used from unripe apples in the trial [27]. AP was separated from the juice using a preparative column with aromatic synthetic adsorbents and a Diaion^®^ HP-20 (Mitsubishi Kasei Co. Ltd., Tokyo, Japan) column. AP samples contained 63.8% procyanidins, which comprised 11.1% dimers, 12.3% trimers, 8.7% tetramers, 5.9% pentamers, 4.9% hexamers, and 20.9% other polymers. AP also contained 12.4% flavan-3-ols, 10.8% hydroxycinnamic acids including chlorogenic acid, and 6.5% phloretin glucosides including phloridzin (Figure S1). The tablets were made with maltitol, agar, and sucrose esters of fatty acids with/without AP. Participants were administered 8 tablets per day for 12 weeks.

### 2.3. Experimental Design

This study was a randomized, double-blind, placebo-controlled trial to evaluate the effects of AP on skin pigmentation induced by UV. The study groups were matched based on age and the MED values determined during the initial health check-up. The experimental design is shown in Table 1.

Participants visited the Dermis Research Center, and the 6th position on the inside of their upper arms was exposed to solar-stimulated UV using a Xenon Arc Solar Simulator with Ushio Optical Module x of UV-A (23 mw/cm^2^) and UV-B (1800 μw/cm^2^) (Ushio Inc., Tokyo, Japan) as the solar light source (0 weeks). At 16–24 h after UV irradiation, skin color was evaluated, and personal MED was determined. After 2 weeks of administering the assigned tablet, skin color lightness (L value) and pigmentation (melanin value) were measured by a spectrophotometer (Konica Minolta Inc., Osaka, Japan) and Mexameter^®^ MX 16 (Courage and Khazaka Electronic GmbH, Köln, Germany) respectively. Then, the 3rd position on the inside of the upper arm was irradiated with 1.5 MED of UV. Skin color lightness and melanin value were measured at 1, 2, 3, 4, 6, and 10 weeks after UV irradiation. The pigmented positions were measured 7 times each time, top and bottom values were deleted, and the average of 3 positions was obtained. To evaluate facial skin conditions, water content and TEWL at the corner of the eye were measured with Skicon-200 EX^®^ (Yayoi Co. Ltd., Tokyo, Japan) and Vapo Scan AS-VT100RS^®^ (Asahi BioMed, Tokyo, Japan), respectively. Facial skin conditions were measured 5 times in an environment testing room with a stable temperature (20 ± 1 °C) and humidity (45 ± 5%) at 0, 4, 8, and 12 weeks. The average value was used.

Strenuous exercise, dietary restrictions, and excessive drinking/eating were restricted during the study period. In addition, ingestion of drugs other than prescribed medications and consumption of polyphenol-rich foods, including green tea, red wine and chocolate, supplements including anti-oxidative additives (vitamin C, CoQ10, etc.), and polyphenols additives (grape seeds, green tea, etc.) that could potentially affect anti-oxidative activity were prohibited during the study period.

### 2.4. Ethics Statement

This study was conducted at the Dermis Research Center Co., Ltd. (Osaka, Japan) in accordance with the Declaration of Helsinki. The study protocol was approved by the Human Study Ethics Committee of Hout Schritt Co. Ltd. (Permit Number 18372). All study participants provided informed content. This study was registered with the University Hospital Medical Information Network Clinical Trials Registry (UMIN-CTR; Japan), number UMIN000039612.

### 2.5. Determination of SOD-like Activity of Apple Procyanidins

Apple procyanidins and their individual oligomeric procyanidin fractions were prepared from Applephenon^®^ using a preparative column as previously described [28]. SOD-like activities in the apple procyanidins and their individual fractions were measured using the SOD assay kit WST (Dojindo Molecular Technologies Inc., Gaithersburg, MD, USA) according to the manufacturer’s instructions. The scavenging activity of superoxide radicals generated in the xanthine/xanthine oxidase system was determined by spectrophotometric measurement of the reductants of water-soluble tetrazolium salt, 2-(4-iodophenyl)-3-(4-nitrophenyl)-5-(2,4-disulphophenyl)-2H-tetrazolium, and monosodium salt (WST-1). The 50% inhibitory effective concentration (EC50) was determined as the concentration of sample that inhibited the formation of WST-1 formazan by 50%, and EC50 percentages were given as the mean value of four experiments. One unit of SOD activity was defined as the amount of enzyme having a 50% inhibitory effect on WST-1.

### 2.6. Statistical Analysis

All data are presented as the mean ± standard deviation. Statistical analysis was performed using two-way analysis of variance (ANOVA) followed by the Bonferroni’s multiple comparisons test. All graphs were produced using GraphPad Prism^®^ software version 8 for Macintosh.

## 3. Results

### 3.1. Effects of AP on UV Irradiation Induced Erythema Value

**Figure 1:**
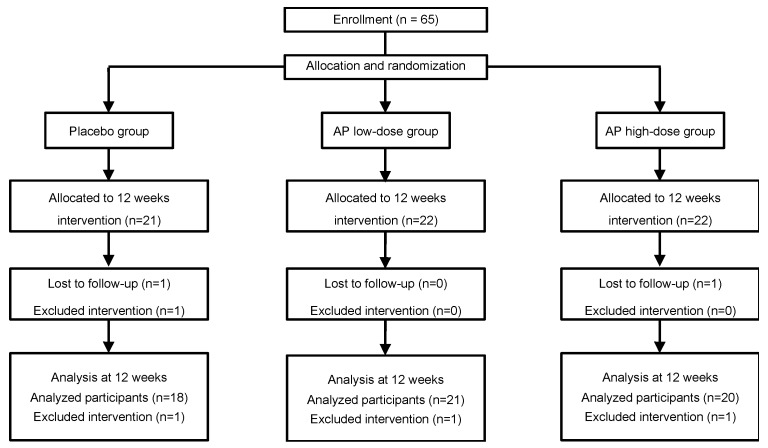
CONSORT (Consolidated Standards of Reporting Trials) diagram. AP: apple polyphenols.

To determine whether AP administration ameliorated the pigmentation caused by UV irradiation, 65 healthy women (age 34.09 ± 4.41 years) were randomly allocated to the placebo and AP groups. However, 3 participants dropped out for personal reasons and 3 participants failed to attend study visits for the measurement, and were therefore excluded from the efficacy analysis. Consequently, the data reported here represent 59 participants (Figure 1).

The baseline characteristics for the placebo and AP groups are shown in Table 2. At baseline, no significant differences were observed between the placebo and the AP groups with regards to any of the skin color parameters, age, MED, water content, and TEWL. All participants had an average >95% ingestion rate. No statistically significant differences were observed for the ingestion rate between the groups. No adverse events related to AP administration were observed during the study period.

To evaluate the effects of AP administration, each skin color parameter measured at the 12-week follow-up was compared between the placebo and the AP groups (Table 3). The effect of AP on UV-induced erythema value, which rapidly increased 24–48 h after UV-irradiation, was evaluated during the study period. In the placebo group, erythema value of pre-irradiation at week 2 significantly increased to 252.0 ± 40.3 from 175.6 ± 34.4 at 1 week after irradiation (*p* < 0.01) and remained significantly higher until week 8. Similarly, in the low-dose and high-dose AP groups, pre-irradiation erythema values (182.4 ± 32.0 and 179.5 ± 51.0) significantly increased to 225.5 ± 34.4 and 228.7 ± 43.5 after 1 week, respectively (*p* < 0.01). However, no significant differences were observed between the placebo and the AP groups during the study period. 

**Figure 2:**
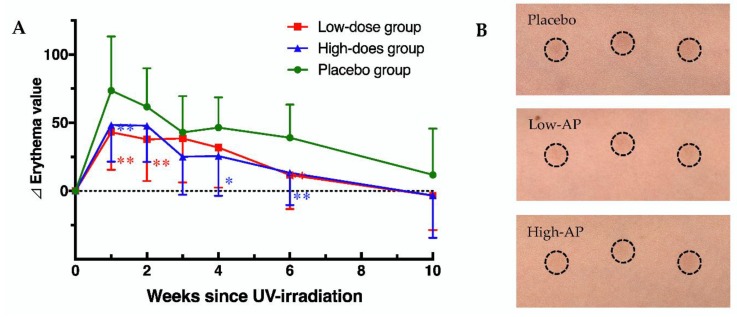
Apple polyphenol (AP) administration prevents increase in the erythema value. (**A**) Changes in erythema value in the placebo and AP groups. (**B**) Representative imaging of an irradiated area in the placebo group (upper) and AP groups (middle and lower) at 1 week after UV irradiation. Data are shown as means ± standard deviation. Significant differences among the groups were determined using repeated-measures of two-way ANOVA followed by Dunnett’s multiple comparisons test (* *p* < 0.05, ** *p* < 0.01).

In contrast, the changes in erythema from baseline (∆ erythema) value of the AP groups were significantly lower than that of the placebo group (Figure 2). Representative imaging of an irradiated area in the placebo group (upper) and AP groups (middle and lower) at 1 week after UV irradiation are shown in Figure 2. These results suggested that oral administration of AP inhibited erythema formation caused by UV irradiation. 

### 3.2. Effects of AP on UV Irradiation Induced Melanin Value

**Figure 3:**
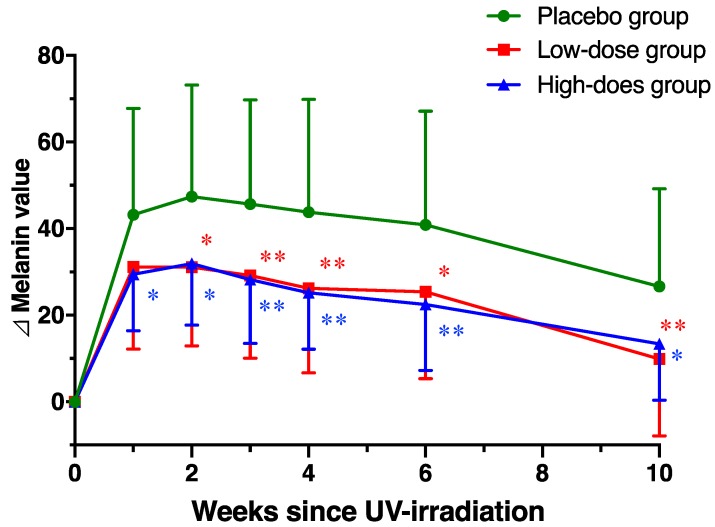
Apple polyphenol (AP) administration prevents increase in the melanin value. Changes in melanin value in the placebo and AP groups. Data are shown as means ± standard deviation. Significant differences among the groups were determined using repeated-measures of two-way ANOVA followed by the Dunnett’s multiple comparisons test (* *p* < 0.05, ** *p* < 0.01).

Next, we evaluated the effect of AP administration on melanin value, which was the pigmentation after UV irradiation. In the placebo group, the melanin value observed was 149.0 ± 33.3 in the skin of the upper arm at week 2. At week 1 to 2 after UV irradiation, the melanin values were significantly increased to 192.2 ± 36.5 and 196.4 ± 38.4, respectively (Table 3). Significantly high melanin values were ongoing at 6 weeks compared the baseline. Similarly, in the low-dose and high-dose AP groups, pre-irradiation melanin values (163.8 ± 29.5 and 159.1 ± 36.6) were significantly increased to 194.9 ± 27.3 and 188.6 ± 33.6 at 1 week after the irradiation, respectively (*p* < 0.05). However, no significant differences were observed between the placebo and the AP groups during the study period. In contrast, the changes in melanin from baseline (∆ melanin) value of the AP groups were significantly lower compared with the placebo group, and the AP groups were protected from the pigmentation induced by UV irradiation (Figure 3).

### 3.3. Effects of AP on UV Irradiation Induced L Value

**Figure 4:**
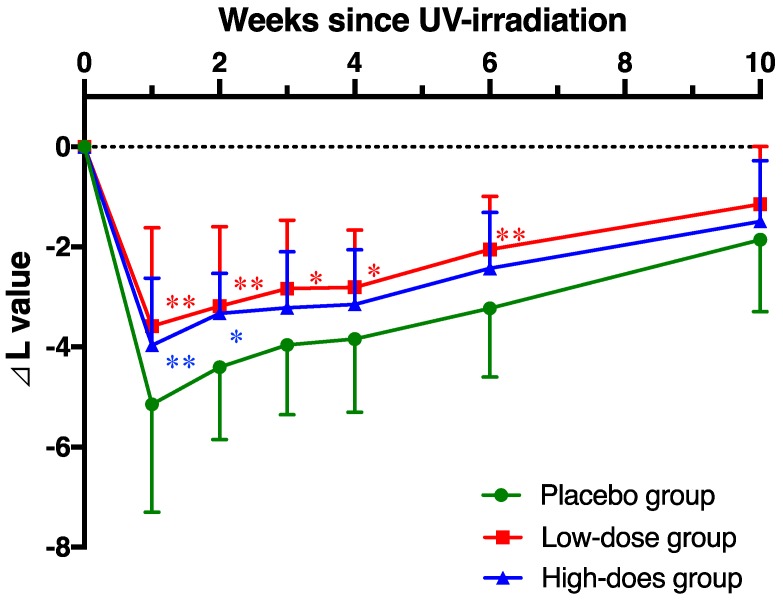
Apple polyphenol (AP) administration prevents decrease in the L value. Changes in L value in the placebo and AP groups. Data are shown as means ± standard deviation. Significant differences among the groups were determined using repeated-measures of two-way ANOVA followed by Dunnett’s multiple comparisons test (* *p* < 0.05, ** *p* < 0.01).

The effect of AP administration on L value (skin lightness) by UV irradiation was evaluated. In the placebo group, L value was observed 64.4 ± 2.4 in the skin of the upper arm at week 0. At week 1 after UV-irradiation, L value was significantly decreased to 59.3 ± 2.7 (*p* < 0.01) and remained significantly lower until week 8 (Table 3). Similarly, in the low-dose and high-dose AP groups, L values of pre-irradiation (64.4 ± 1.9 and 64.7 ± 2.7) were significantly decreased to 60.8 ± 2.4 and 60.4 ± 2.0 at 1 week after the irradiation, respectively (*p* < 0.05). L value reduction in the AP groups was less than the placebo group, but there were no significant differences between the placebo and AP groups during the study period. In contrast, the decrease in L value from the baseline (∆ L) of the AP groups was significantly inhibited compared with the placebo group, and AP administration was protected from the reduction of skin lightness induced by UV irradiation (Figure 4), although a dose-dependent relationship was not clearly observed.

### 3.4. Effect of AP on Facial Skin Condition

To evaluate the effects of AP dietary supplements, water content and TEWL in facial skin were measured at the 12-week follow-up and compared between the placebo and the AP groups. No significant differences were detected between the groups for water content and TEWL (Table 4). AP intervention had no significant effect on any of the other facial skin condition parameters measured.

### 3.5. Effect of Apple Procyanidins on SOD-Like Activity

Apple procyanidin and their individual apple procyanidin fractions were prepared using normal-phase HPLC. All of the oligomer fractions strongly inhibited the generation of superoxide radicals. The EC50 values of individual fractions from monomer to hexamer are as follows: monomer, 4.53 µM (1.32 µg/mL); dimer, 2.60 µM (1.51 µg/mL); trimer, 1.88 µM (1.63 µg/mL); tetramer, 1.82 µM (2.10 µg/mL); pentamer, 2.25 µM (3.25 µg/mL); and hexamer, 2.33 µM (4.03 µg/mL). The superoxide radical scavenging activities varied in the following order: tetramer > trimer > pentamer > hexamer > dimer > monomer. EC50 value of apple procyanidins was 2.43 µg/mL.

## 4. Discussion

Various physiological functions of procyanidins have been reported, and the data strongly suggest that the antioxidative properties and radical scavenging activities of procyanidins played an important part in the mechanisms underlying their various functionalities [29]. UV exposure results in increased ROSs and free radical generation, which cause damage to proteins, lipids, and DNA [6]. Specifically, ROSs induce DNA damage, such as DNA base damage, and DNA single- and double-strand breaks. UV irradiation modifies DNA by directly forming cyclobutane pyrimidine dimers and 6–4 photoproducts and by indirectly producing 8-OHdG [8]. DNA damage not only induces genetic mutations and cellular apoptosis but also melanocyte proliferation in skin tissue. Similarly, ROSs induce pigmentation of the skin and play an important role in regulating melanogenesis in melanocytes. 8-OHdG [30] and thymine dimers [31] have been reported to stimulate melanogenesis in melanocytes. In our previous study, we reported that apple procyanidin concentrations were significantly correlated with hydrophilic oxygen radical absorbance capacity (H-ORAC) values (r = 0.8284, *p* < 0.0001) in 30 varieties of apple cultivars [25]. SOD-like activity measurement using WST-1 showed that apple procyanidins have strong SOD-like activity, although the relationship between polymerization degree of apple procyanidins was not clearly observed. These data suggest that the anti-oxidative activity of AP mainly is due to procyanidins. The current study indicated that apple procyanidins protected against the oxidative stress caused by UV irradiation and inhibited melanin synthesis in melanocytes.

Procyanidins, derived from grape seeds, pine bark, and cacao, are reported to have strong protective effects against ROSs and free radicals [32,33,34]. Oral administration of French maritime pine bark extracts, which are rich in flavonoids such as flavan-3-ols and procyanidins, protected against UV-induced skin damage [35], decreased clinical grading of skin photo-aging scores [36], and clinically improved the melasma [37]. However, due to the low numbers of participants and the lack of placebo groups in some papers, the effects of pine bark procyanidin on skin pigmentation are not fully understood and further study is required. In the current study, we demonstrated that AP oral administration, specifically procyanidins, was effective in inhibiting pigmentation by UV irradiation in this human clinical study. The results suggested that the reduction in oxidative stress and the antioxidative activity by AP resulted in the inhibition of melanogenesis induced by UV irradiation, which is consistent with previous studies [36,37]. In our previous study, we reported that AP inhibited mushroom tyrosinase and the generation of melanin pigments in a B16 mouse melanoma cell study in vitro, which was a non-UV irradiation system [26]. Although we obtained limited results for skin color values induced by UV irradiation in high-dose and low-dose AP participants, the current clinical trial is the first study to show the effects of daily administration of AP for 12 weeks on skin pigmentation.

The bioavailability of procyanidins is a prerequisite to understanding their mechanisms in physiological function studies. Animal and human studies suggested that after administration of procyanidins, flavan-3-ols (–)-epicatechin and procyanidin dimers were absorbed, and the conjugated and methylated forms metabolized by detoxification enzymes were detected in the blood [38,39]. We also reported that after the administration of apple procyanidins, up to tetramer was observed in rat plasma using high-performance liquid chromatography/electrospray ionization tandem mass spectrometry (HPLC-ESI/MS) [40]. However, the absorption of procyanidins by the small intestine is very low, and most of the administered procyanidins were degraded in the large intestine by the gut microbiota [41]. Recently, several studies demonstrated that procyanidins, including apple [42], cranberry [43], and grape [44], were associated with a change in the gut microbiota, including a decrease in the Firmicutes/Bacteroidetes ratio, and contributed to the prevention of obesity and diabetes in mice fed a high-fat diet. Additionally, we reported that apple procyanidin administration suppressed inflammation in the liver caused by endotoxic lipopolysaccharides from gram-negative bacteria, and ameliorated the intestinal gut barrier, thereby providing anti-obesity benefits [42]. Some studies have demonstrated how modulation of the gut microbiome can influence skin homeostasis pathways to counteract UV damage [45]. AP administration might therefore influence the suppression of pigmentation and oxidation through the gut microbiota.

In the present study, the 12-week intervention consisting of AP showed no significant difference in facial skin parameters (water content and TEWL) at any dose. Facial skin parameters are greatly affected by the sexual cycle and lifestyle. Participants did not control their UV exposure in facial skin because they were living normally during the study. Grape seed procyanidin was reported to protect the impaired skin structures induced by UV due to the inhibition of matrix metalloproteinase in an animal model [46]. Human intervention studies with controlled UV exposure are required to determine whether AP influences skin pigmentation and to evaluate the effects of chronic AP administration on skin parameters. Since procyanidins are predominant in modern diets, including those found in fruits, cacao, tea, and red wine to name few, it is important to further clarify their effects on skin conditions. We anticipate that our findings may be useful for future pharmacotherapy development, clinical practice, and the identification and characterization of functional foods.

## Figures and Tables

**Table 1:** Trial study design.

**Intervention Period (Weeks)**	**0**	**2**	**3**	**4**	**5**	**6**	**8**	**12**
**(Weeks since UV Irradiation)**		**(0)**	**(1)**	**(2)**	**(3)**	**(4)**	**(6)**	**(10)**
Precheck	●							
Visit to facility	●	●	●	●	●	●	●	●
Informed consent	●							
MED evaluation	●							
Evaluation of pigmentation								
UV-irradiation (1.5 MED)		●						
Erythema, melanin, L value		●	●	●	●	●	●	●
Evaluation of facial skin condition								
Water content, TEWL	●			●			●	●
Ingestion of test sample	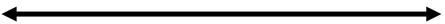							
Recording in diary	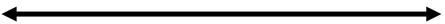							

MED: minimal erythema dose; TEWL: trans-epidermal water loss. ●: visits for the measurement.

**Table 2:** Baseline (0 weeks) characteristics of study participants.

	Apple Polyphenol Groups		Placebo Group
	Low-Dose Group (300 mg/day)	High-Dose Group (600 mg/day)	
N	21	20	18
Age (y)	34.19 ± 4.29	35.20 ± 3.89	34.06 ± 4.08
MED (mJ/cm^2^)	5323.7 ± 478.0	5471.1 ± 499.7	5247.0 ± 531.7
Water content (AU)	88.56 ± 18.62	80.94 ± 14.33	85.32 ± 20.59
TEWL (g/h m^2^)	15.80 ± 5.85	16.12 ± 5.58	14.92 ± 5.10

Values are means ± standard deviation. None of the characteristics was different among the groups at baseline by the Tukey’s multiple comparisons test. MED: minimal erythema dose; TEWL: trans-epidermal water loss.

**Table 3:** Effects of apple polyphenol (AP) on pigmentation induced by UV irradiation.

Parameters	Groups	Intervention Period (Weeks since UV-Irradiation)						
		0	1	2	3	4	6	10
Erythema value	Placebo	175.6 ± 34.4	252.0 ± 40.3 **	237.3 ± 36.9 **	219.5 ± 34.7 **	222.5 ± 38.5 **	212.8 ± 31.9 *	187.6 ± 35.4
	Low AP	182.4 ± 32.0	225.5 ± 34.4 **	222.5 ± 31.9 **	224.0 ± 40.0 **	215.9 ± 41.5 *	196.6 ± 31.8	184.0 ± 26.6
	High AP	179.5 ± 51.0	228.7 ± 43.5 **	225.0 ± 52.5 **	204.6 ± 40.0	206.1 ± 36.6	193.4 ± 42.8	176.2 ± 40.3
Melanin value	Placebo	149.0 ± 33.3	192.2 ± 36.5 **	196.4 ± 38.4 **	194.7 ± 37.4 **	192.9 ± 36.8 **	189.9 ± 35.8 **	175.7 ± 32.1
	Low AP	163.8 ± 29.5	194.9 ± 27.3 *	194.9 ± 28.2 *	193.0 ± 27.2 *	190.0 ± 28.5 *	189.2 ± 28.7	174.5 ± 23.3
	High AP	159.1 ± 36.6	188.6 ± 33.6 *	191.1 ± 33.7 **	187.4 ± 31.7	184.3 ± 33.5	181.6 ± 33.2	172.5 ± 31.0
L value	Placebo	64.4 ± 2.4	59.3 ± 2.7 **	60.0 ± 2.1 **	60.4 ± 2.0 **	60.6 ± 2.5 **	61.2 ± 2.1 **	62.5 ± 2.1
	Low AP	64.4 ± 1.9	60.8 ± 2.4 **	61.2 ± 2.1 **	61.5 ± 1.8 **	61.6 ± 1.8 **	62.3 ± 1.6 *	63.2 ± 1.9
	High AP	64.4 ± 2.7	60.4 ± 2.0 **	61.0 ± 2.5 **	61.1 ± 2.1 **	61.2 ± 2.0 **	61.9 ± 2.2 **	62.9 ± 2.2 **

Values are means ± standard deviation. Significant differences within the groups were determined using repeated-measures of two-way ANOVA followed by the Dunnett’s multiple comparisons test (** *p* < 0.01, * *p* < 0.05).

**Table 4:** Effects of apple polyphenol (AP) on water content and trans-epidermal water loss (TEWL) in facial skin.

Parameters	Groups	Intervention Period (Weeks)			
		0	4	8	12
Water content (AU)	Placebo	85.3 ± 20.6	89.6 ± 16.6	88.0 ± 16.9	92.0 ± 16.8
	Low AP	88.6 ± 18.6	92.8 ± 16.6	91.8 ± 22.5	92.2 ± 15.8
	High AP	80.9 ± 14.3	86.7 ± 14.6	88.3 ± 13.9	91.3 ± 12.3
TEWL (g/h m^2^)	Placebo	14.9 ± 5.1	14.6 ± 5.3	15.1 ± 6.2	13.6 ± 3.7
	Low AP	15.8 ± 5.9	17.0 ± 6.9	15.9 ± 7.5	15.4 ± 7.6
	High AP	16.1 ± 5.6	16.2 ± 4.1	14.5 ± 4.3	14.8 ± 6.4

Values are means ± standard deviation.

## Supplementary Materials

The following are available online at https://www.mdpi.com/2072-6643/12/4/1071/s1, Figure S1: Chemical structures of major apple polyphenols.
