# Supplementary material for: Administration of Apple Polyphenol Supplements for Skin Conditions in Healthy Women: A Randomized, Double-Blind, Placebo-Controlled Clinical Trial
Source: Nutrients. 2020 Apr 13;12(4):1071. doi: 10.3390/nu12041071 (PMC7231294; doi:10.3390/nu12041071)
Supplement: Supplementary file 1 [file nutrients-12-01071-s001.pptx]

## Slide 1
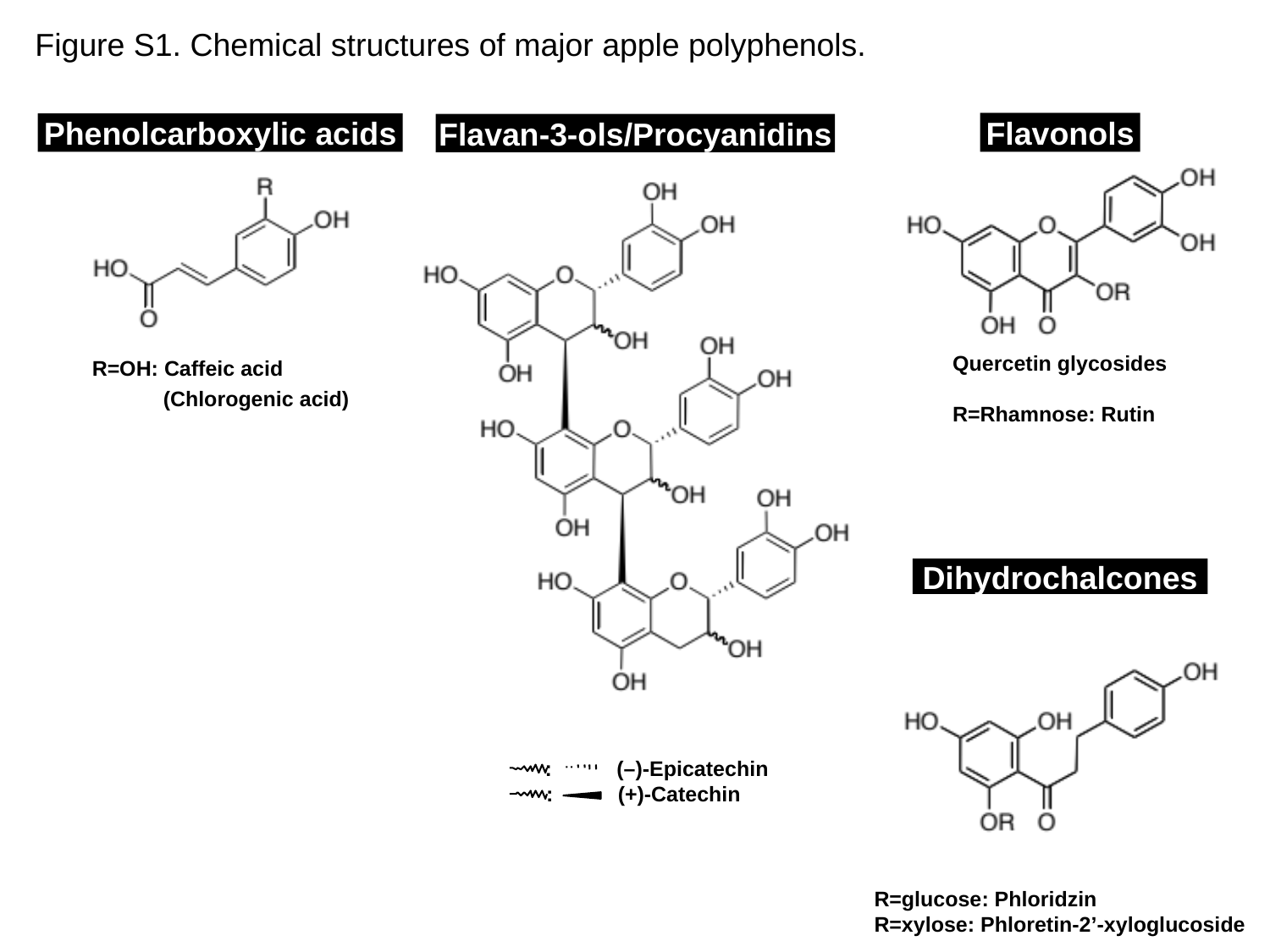

Figure S1. Chemical structures of major apple polyphenols.
Flavonols
Phenolcarboxylic acids
Flavan-3-ols/Procyanidins
R=OH: Caffeic acid
 (Chlorogenic acid)
Quercetin glycosides
R=Rhamnose: Rutin
 Dihydrochalcones
: (–)-Epicatechin
: (+)-Catechin
R=glucose: Phloridzin
R=xylose: Phloretin-2’-xyloglucoside
